# Lunasin Alleviates Allergic Airway Inflammation while Increases Antigen-Specific Tregs

**DOI:** 10.1371/journal.pone.0115330

**Published:** 2015-02-03

**Authors:** Xiaowei Yang, Jingjing Zhu, Chun-Yu Tung, Gail Gardiner, Qun Wang, Hua-Chen Chang, Baohua Zhou

**Affiliations:** 1 Department of Pediatrics, HB Wells Center for Pediatric Research, Indiana University School of Medicine, Indianapolis, IN, 46202, United States of America; 2 Department of Microbiology and Immunology, Indiana University School of Medicine, Indianapolis, IN, 46202, United States of America; 3 Department of Veterinary Medicine, Southwest University at Rongchang, Rongchang, China; 4 Department of Biology, School of Science, Indiana University Purdue University at Indianapolis, Indianapolis, IN, 46202, United States of America; Baylor Institute for Immunology Research, UNITED STATES

## Abstract

Lunasin is a naturally occurring peptide isolated from soybeans and has been explored in cancer treatment. Lunasin inhibits NF-κB activation and thus pro-inflammatory cytokine and mediator production in macrophages. In this study we demonstrate that lunasin can effectively suppress allergic airway inflammation in two murine models of asthma. In an OVA+Alum sensitization model, intranasal lunasin treatment at the time of OVA challenges significantly reduced total cells counts in bronchoalveolar lavage (BAL) fluid and eosinophilia, peribronchiolar inflammatory infiltration, goblet cell metaplasia and airway IL-4 production. In an OVA+LPS intranasal sensitization model, lunasin treatment either at the time of sensitization or challenge has similar effects in suppress allergic airway inflammation including significantly reduced total cell and eosinophil counts in BAL fluid, inflammatory gene *Fizz1* expression in the lung, and IL-4 production by OVA re-stimulated cells from mediastinal lymph nodes. We further show that intranasal instillation of OVA+lunasin significantly increases OVA-specific regulatory T cell (Treg) accumulation in the lung comparing to OVA only treatment. Taken together, our results suggest lunasin as an anti-inflammatory agent can be potentially used in asthma therapy or as an adjuvant to enhance the induction of antigen-specific Tregs and thus boost the efficacy of allergy immunotherapy.

## Introduction

Prevalence of asthma has increased over the past 2–3 decades affecting 5–10% of the population in many developed countries and is associated with a large socioeconomic burden [1]. Majority of asthma is associated with dysregulated T helper type-2 (Th2) immunity leading to chronic eosinophilic inflammation in the airways. Recent studies demonstrated that asthma is rather heterogeneous and complex in pathogenesis involving wide range of innate and adaptive immune cells and cytokines [2]. Treatments for asthma have been improved substantially in recent years. However, current treatments such as antihistamines, leukotriene receptor antagonists and glucocorticoids are effective in temporarily relieve symptoms by suppressing inflammation but do not change the chronic course of disease [3]. Furthermore, these therapies failed to control the symptoms in 5–10% severe persistent asthma patients. New and more effective therapies are urgently required.

Induction of antigen-specific regulatory T (iTreg) cells is an important mechanism to maintain mucosal tolerance against harmless antigens and dampen the on-going allergic inflammation at exacerbation in animal models [4–7]. Induction of allergen-specific iTregs in allergic patients is an essential step in allergen-specific immunotherapy and associates with clinical efficacy and the suppression of allergic inflammation [8–10]. With the advantages of long-term allergen-specific immune tolerance and disease-modifying effects, allergen-specific immunotherapy is the only available curative treatment for allergic diseases.

Lunasin was originally isolated from soybeans, and is a naturally occurring peptide containing 43 amino acids [11]. This peptide exhibits properties that have multiple health benefits, and is a promising chemopreventive agent [12, 13] and therapeutic agent for cancer [14–17]. Studies demonstrate that lunasin inhibits NF-κB pathway and thus the production of proinflammatory cytokines and mediators by macrophages [18–20]. To further define the functions of lunasin, we determine its effects on modulating allergic inflammation in the preclinical study of asthma models. Allergic asthma was induced in BALB/c mice following systemic sensitization with Alum-adjuvanted ovalbumin (OVA). Intransal administration of lunasin while challenging with OVA alleviated allergic airway inflammation as evidenced by reduced eosinophilic inflammatory cell infiltration in the bronchoalveolar lavage (BAL) and airway goblet cell metaplasia. In an additional asthma model induced by intranasal LPS, administration of lunasin at either sensitization or challenge reduced airway inflammation. Attenuation of allergic inflammation following lunasin treatment was associated with increased numbers of OVA-specific iTregs (CD4^+^ KJ1.26^+^ Foxp3^+^) in the lung. Collectively, these studies suggest a potential treatment with administration of lunasin to reduce allergic inflammation with added benefit of promoting the accumulation of allergen-specific iTregs in airways which could have long-lasting effects to dampen allergic responses.

## Materials and Methods

### Mice and Ethics Statement

BALB/c mice were purchased from Jackson Laboratory and maintained in pathogen-free conditions. All studies were approved (protocol 10352) by the Indiana University School of Medicine Animal Care and Use Committee.

### Antibodies, lunasin peptide, and other reagents

Fluorochrome-conjugated monoclonal antibodies to mouse CD4, KJ1.26, IL-4, and Foxp3 were obtained from Biolegned. IL-4 ELISA kit was purchased from Biolegend. The lunasin peptide with 43-amino acid was chemically synthesized with 97% purity by LifeTein (South Plainfield, NJ) [16]. Ovalbumin (OVA, chromatographically purified) was from Worthington Biochemical Corp (Lakewood, NJ).

### OVA+Alum sensitized allergic airway inflammation

BALB/c mice (female, 6–8 weeks old) were sensitized twice with intraperitoneal (i.p.) injection of OVA (100 µg) adsorbed in Alum at days 0 and 7. One week following the second sensitization, mice were challenged with intranasal (i.n.) instillation of 100 µg of OVA alone or with lunasin (20 µg/mouse) in a volume of 40 µl daily for 3 consecutive days (at days 14–16). Control mice were treated with PBS only. One day after the last challenge (day 17), animals were euthanized for analysis.

### OVA+LPS sensitized allergic airway inflammation

OVA+LPS sensitization were performed as previously described [21]. BALB/c mice (female, 6–8 weeks old) were sensitized three times with i.n. instillation of OVA (100 µg) mixed with LPS (50 ng) in a volume of 40 µl at days 0, 1, and 2. Mice were challenged with i.n. instillation of OVA (100 µg) in a volume of 40 µl at days 14, 15, 18, and 19. One day after the last challenge (at day 20), animals were euthanized for analysis.

The group of mice received lunasin at the time sensitization, 20 µg lunasin in 200 µl PBS were injected intraperitoneally (i.p.) on days 0–2. For the group received lunasin at challenge, 20 µg lunasin were mixed with 100 µg OVA in 40 µl PBS and administered intranasally on days 14, 15, 18 and 19.

### Analysis of allergic airway inflammation

The bronchoalveolar lavage (BAL) fluid was obtained to determine the total cell counts. The number for eosinophils was analyzed on cytospin slide after stained with a modified Wright-Giemsa stain on a Hematek 2000 slide stainer (Bayer) [21, 22]. Intracellular cytokine staining was performed in CD4^+^ T cells from mediastinal lymph nodes (MedLN) as previously described [21, 22]. IL-4 concentration in BAL fluid and media from MedLN cells cultured with 100 µg/ml OVA for three days was determined by ELISA.

Left side two lobes of the lungs were homogenized in Trizol reagent to extract RNA and gene expression was determined by Taqman Realtime PCR (Life Technologies). Right side three lobes of the lungs were fixed in neutral buffered formalin. Paraffin embedded sections were stained with hematoxylin and eosin (H&E) or Periodic-acid Schiff (PAS) to evaluate the infiltration of inflammatory cells and goblet cell metaplasia of the airways.

### Adoptive cell transfer and OVA-specific iTreg induction

Naïve OVA-specific CD4^+^ T cells (KJ1.26^+^) were purified from DO11.10 transgenic mice expressing TCRs specific for an MHC class II restricted epitope of OVA_323–339_ (The Jackson Laboratory). Two millions of naïve KJ1.26^+^ CD4^+^ T cells were adoptively transferred into BALB/c mice. The following day these mice received daily i.n. instillation of 100 µg OVA in the presence or absence of 20 µg lunasin for 3 consecutive days. One day after the last instillation, animals were euthanized to collect the mediastinal lymph nodes and lungs for analysis of OVA-specific iTregs by staining with Foxp3 expression.

## Results

### Lunasin attenuates allergic airway inflammation in mice sensitized with OVA+Alum

**Figure 1:**
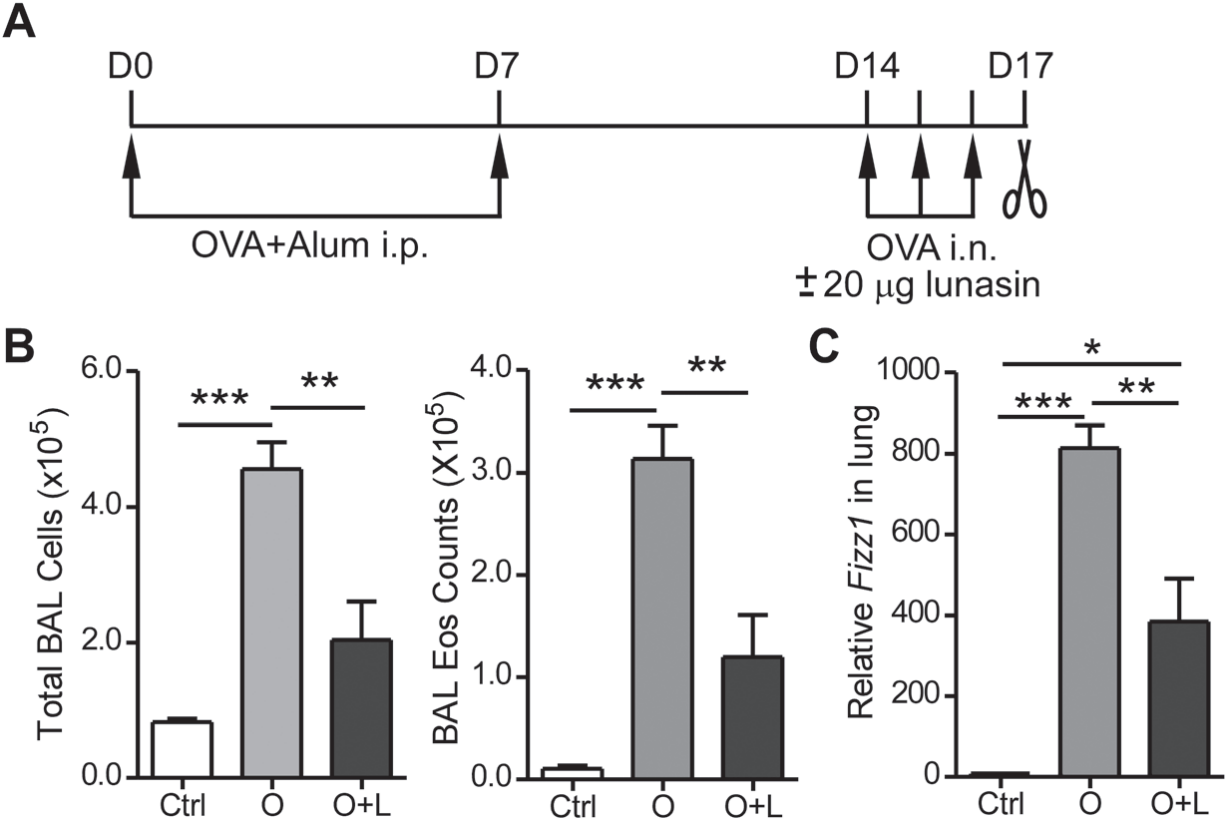
Lunasin alleviates allergic airway inflammation. (A) Experimental procedures. BALB/c mice were sensitized intraperitoneally (i.p.) with 100 µg OVA absorbed on 1.3 mg Alum on days 0 and 7. Animals were challenged intranasally with 100 µg OVA in the presence or absence of 20 µg lunasin on days 14 to 16 and sacrificed on day 17 for analysis. Control mice were treated similarly with PBS. (B) Total and eosinophil cell counts in bronchoalveolar lavage (BAL) fluid. (C) *Fizz1* gene expression in the lungs determined by realtime PCR. Ctrl: PBS treated mice; O: OVA treated mice; O+L: OVA plus lunasin treated mice. Data represent mean ± SEM (n = 5). *: p < 0.05; ** p < 0.01; ***: p < 0.001 by one-way analysis of variance with Bonferroni’s post-hoc tests.

Lunasin exhibited anti-inflammatory activity through inhibiting NF-κB pathway in macrophages [18, 19]. To study the effects of lunasin in suppressing allergic airway inflammation, we sensitized BALB/c mice with OVA and Alum followed by intranasal (i.n.) OVA challenge (Fig. 1A). 20 µg lunasin was mixed with 100 µg OVA to intranasally challenge the treatment group of mice while control group was treated OVA or PBS only. The mice treated with OVA+lunasin had significantly fewer total inflammatory cell counts in the BAL with reduced eosinophils compared with OVA treated mice (Fig. 1B). Gene found in inflammatory zone 1 (*Fizz1*) encodes a cysteine-rich secreted protein associated with pulmonary inflammation and is capable of promoting expression of α-SMA and type I collagen at the early stages of airway remodeling in murine model of asthma [23]. Although significantly higher than the PBS treated controls, mice received OVA+lunasin treatment had significantly reduced *Fizz1* gene expression in their lungs than the OVA only challenged mice, (Fig. 1C).

**Figure 2:**
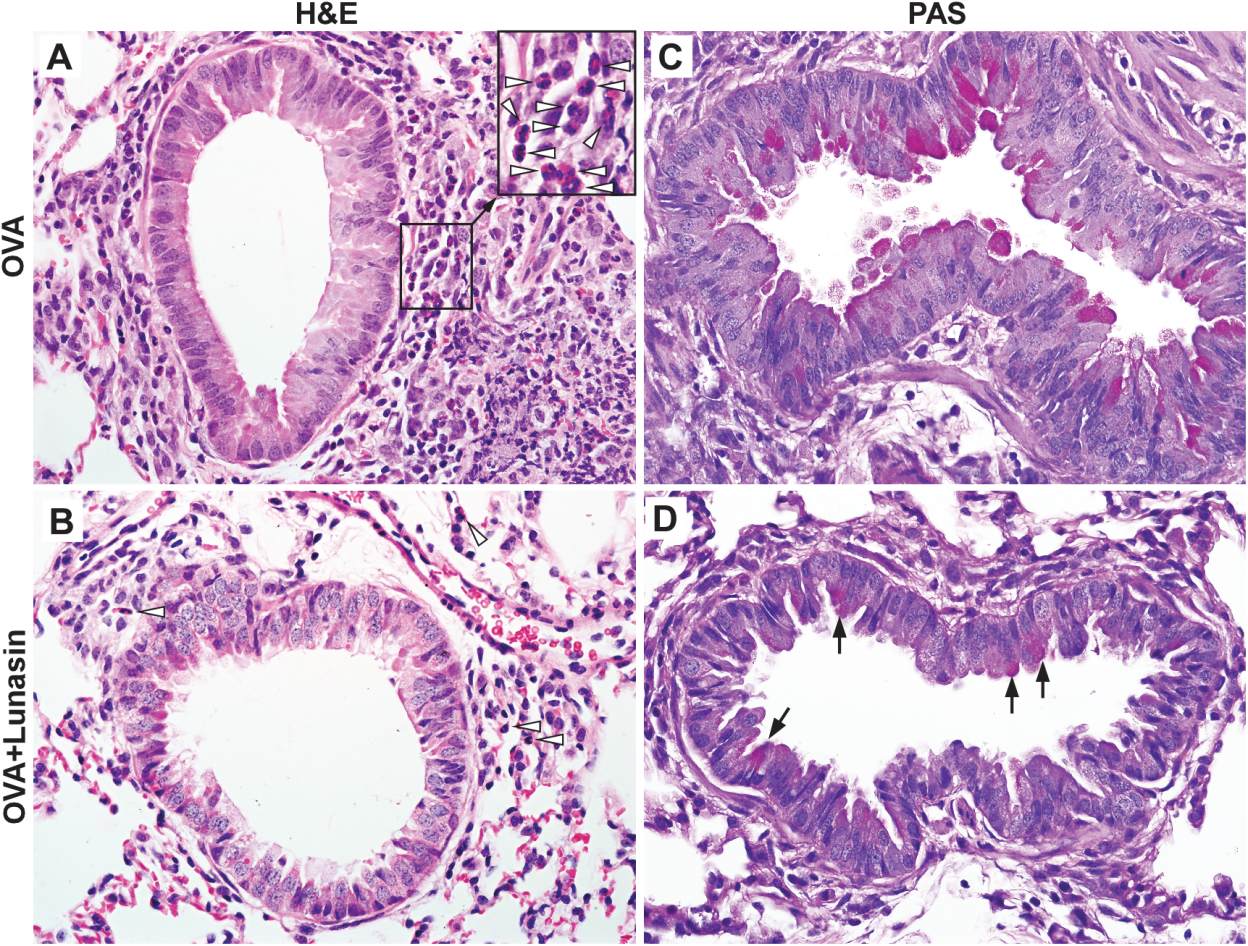
Lunasin reduces eosinophilic infiltration and goblet cell metaplasia. Mice were sensitized and challenged as described in Fig. 1A. (A and B) Hematoxylin and eosin (H&E) stained lung sections from OVA (A) or OVA+lunasin treated (B) mice. Open arrow head: eosinophils. (C and D) Periodic acid–Schiff (PAS) stained lung sections from OVA (C) or OVA+lunasin treated (D) mice. Arrow: mucus containing goblet cells.

Consistent to reduced pulmonary *Fizz1* gene expression (Fig. 1C), histological study revealed that lungs from control mice exhibited severe peribronchiolar eosinophilic infiltration (Fig. 2A) whereas lunasin treated mice had greatly reduced inflammatory infiltration with scattered eosinophils (Fig. 2B). One of the cardinal features of human asthma is airway goblet cell metaplasia and mucus hyperproduction. While lungs from all the control mice had mild to modest goblet cell metaplasia (Fig. 2C), only 2 out of 5 lunasin treated mice showed mild goblet cell metaplasia in the airways (arrows in Fig. 2D).

**Figure 3:**
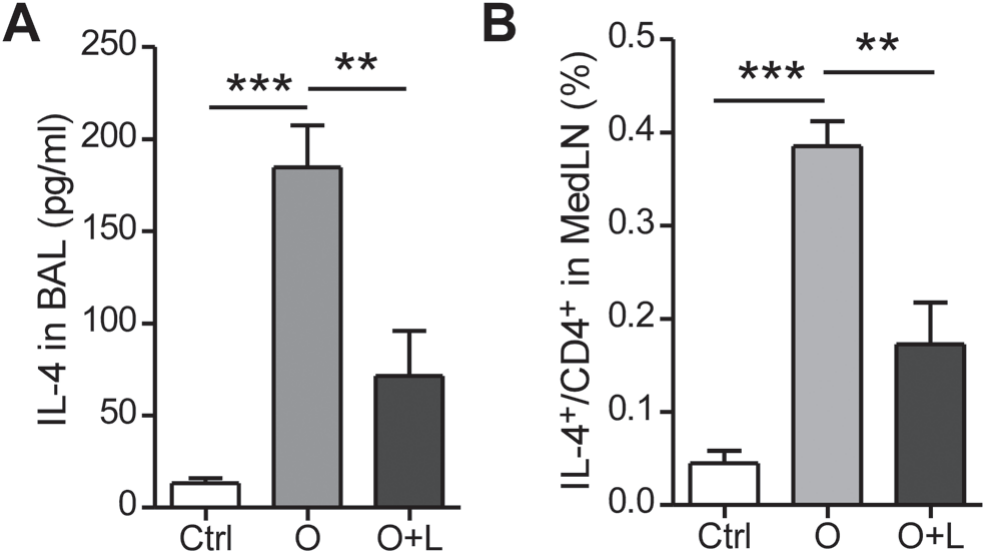
Lunasin reduces IL-4 expression in the airways. Mice were sensitized and challenged as described in Fig. 1A. (A) IL-4 concentration in BAL fluid determined by ELISA. (B) Percentage of IL-4^+^ in CD4^+^ T cells in the mediastinal lymph nodes (MedLN) determined by intracellular staining. Data represent mean ± SEM (n = 5). **: p < 0.01; ***: p < 0.001 by one-way analysis of variance with Bonferroni’s post-hoc tests.

Allergic airway inflammation is characterized with increased Th2 cytokines in the airways. We measured IL-4 concentration in the BAL fluid by ELISA and found BAL IL-4 concentration was reduced from ~185 pg/ml in OVA treated mice to ~ 70 pg/ml in OVA+lunasin treated mice (Fig. 3A). When lung draining mediastinal lymph node cells were restimulated and stained for IL-4 expression, lunasin treated mice had significantly less IL-4^+^CD4^+^ cells compared to control mice (Fig. 3B).

### Lunasin alleviates allergic airway inflammation in OVA+LPS sensitized mice

**Figure 4:**
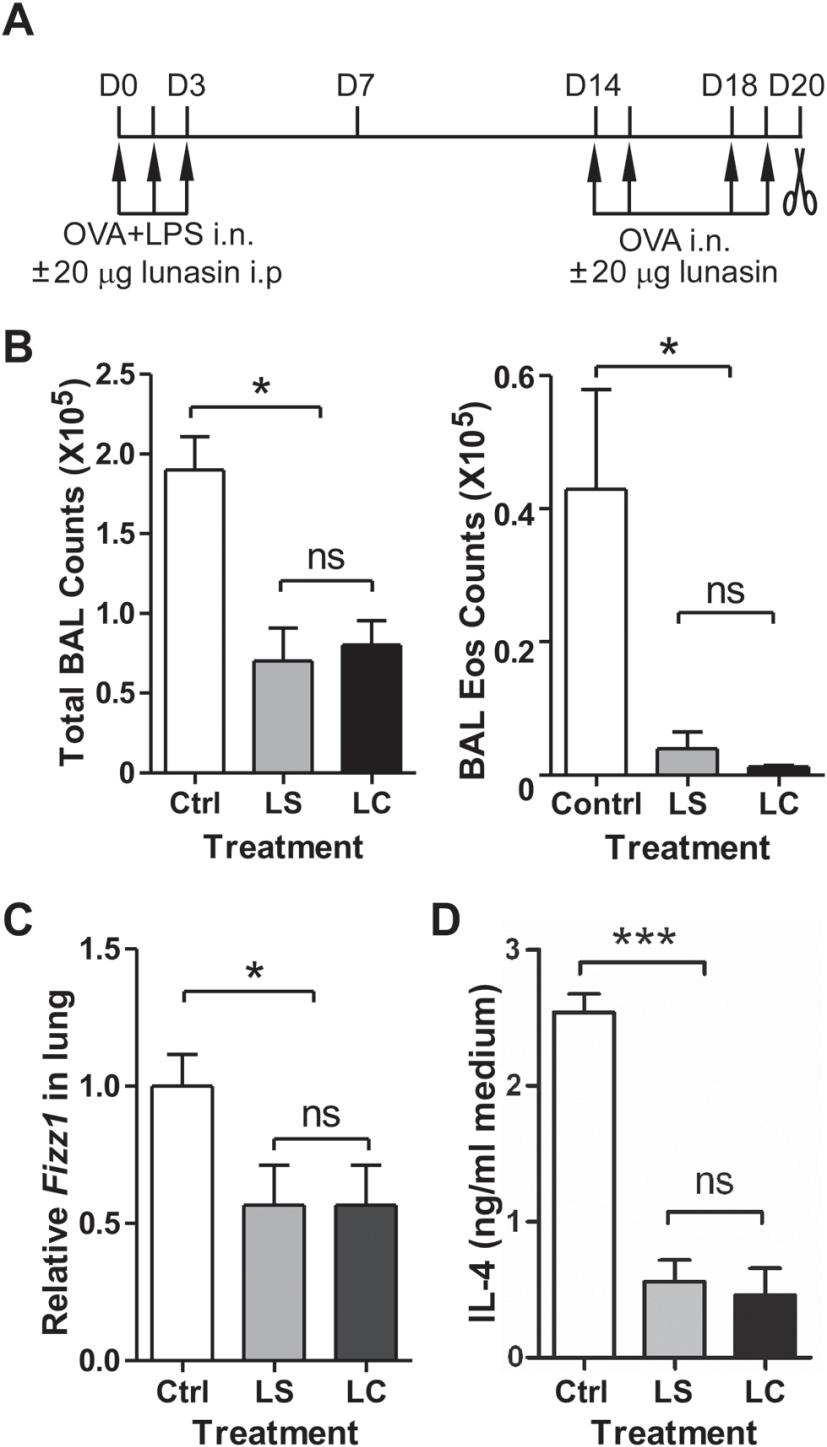
Lunasin alleviates allergic airway inflammation in an intranasal LPS sensitization model. (A) Experimental procedures. BALB/c mice were sensitized intranasally with 100 µg OVA + 50 ng LPS on days 0 to 3. Mice were challenged with 100 µg OVA on days 14, 15, 18, and 19 and sacrificed on day 20 for analysis. Lunasin (20 µg/mouse) were given either intraperitoneally (i.p.) at sensitization or intranasally (i.n.) at challenge. (B) Total and eosinophil cell counts in BAL fluid. (C) *Fizz1* gene expression in the lungs determined by realtime PCR. (D) IL-4 concentration in media after cells from mediastinal lymph nodes were cultured with 100 µg OVA for 3 days. Ctrl: Control mice; LS: group of mice received lunasin at sensitization; LC: group of mice received lunasin at challenge. Data represent mean ± SEM (n = 5). *: p < 0.05; *** p < 0.001 by one-way analysis of variance with Bonferroni post-hoc test.

The OVA+Alum model is a well-defined mouse asthma model resulting in Th2-mediated allergic airway inflammation with many characteristics of asthma including airway eosinophilia, mucus hyperproduction, and airway hyperresponsiveness [24, 25]. However, immunization with OVA+Alum to induce sensitization is not a physiological route of allergen exposure. Sensitization with antigen and LPS through the respiratory system, the anatomic site that would normally encounter environmental allergens and LPS, provides a particular relevant model to investigate immune responses in the pathogenesis of asthma [21, 26, 27]. Mice were sensitized by intranasally delivery of 100 µg OVA in the presence of 50 ng LPS on days 0, 1 and 2 (Fig. 4A). To study the effects of lunasin on Th2 sensitization induced by OVA+LPS, one group of mice also received intraperitoneal (i.p.) injection of 20 µg lunasin at the same time as the mice were sensitized. Mice were then challenged intranasally with 100 µg OVA on days 14, 15, 18, and 19 in the presence or absence of 20 µg lunasin. Both groups of mice, treated with 20 µg lunasin either i.p. at sensitization or i.n. at challenge, had reduced total inflammatory cell counts with significantly fewer eosinophils in BAL fluid than the control group (Fig. 4B). Likewise, pulmonary expression of *Fizz1* gene in lunasin treated mice was also significantly lower than that in control animals (Fig. 4C). When cultured in 100 µg OVA for three days, the mediastinal lymph node cells isolated from lunasin treated groups produced 0.5 ng/ml IL-4 comparing to 2.5 ng/ml by the cells from control group (Fig. 4D).

### Lunasin promotes antigen-specific iTreg development in airways

**Figure 5:**
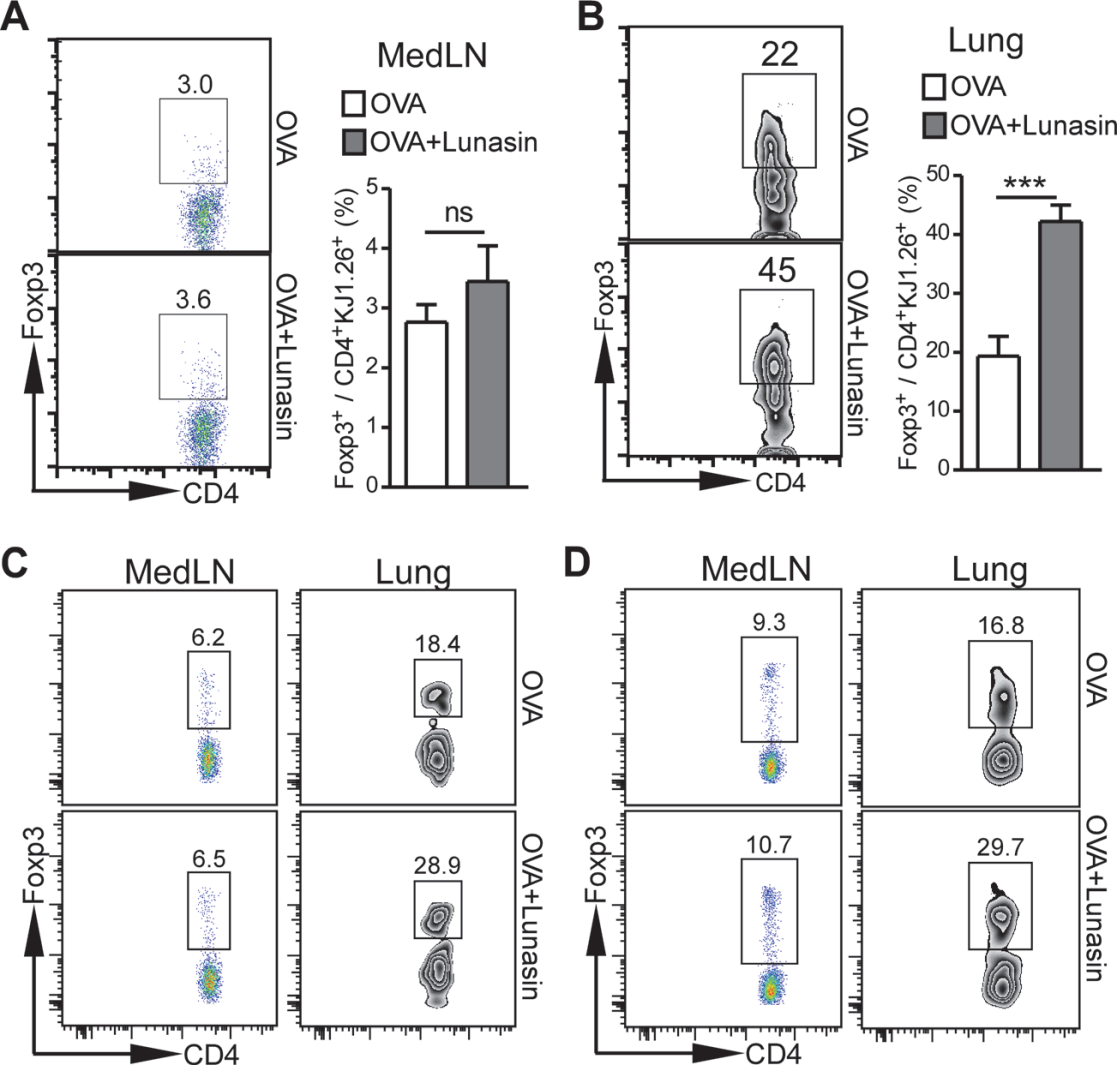
Lunasin promotes antigen-specific iTreg accumulation in the lung. OVA-specific naïve CD4+ T cells were purified from DO11.10 mice and 2 × 10^6^ cells were adoptively transferred into BALB/c mice. The recipient mice were treated with 100 µg OVA intranasally for three days in the presence or absence of 20 µg lunasin. (A) OVA-specific iTregs in mediastinal lymph nodes (MedLN). (B) OVA-specific iTregs in lungs. (C) OVA-specific iTregs in MedLN and lung 7 days after the last intranasal treatment. (D) OVA-specific iTregs in MedLN and lung 14 days after the last intranasal treatment. Data represent mean ± SEM (n = 5). ns: not significant; ***: p < 0.001 by Student’s *t* test.

Induction of antigen-specific iTregs controls the severity of OVA-induced chronic airway inflammation [4] while blockade of iTreg development in neonatal mice maintains exaggerated responses to inhaled house dust mite through to adulthood [28]. Since intranasal lunasin treatment at the time of challenge dampens allergic airway inflammation in both OVA+Alum (i.p.) and OVA+LPS (i.n.) sensitized mice, we hypothesize that lunasin can promote antigen-specific iTreg development in the airways. To test this hypothesis, we purified OVA-specific naïve CD4^+^ T cells from DO11.10 mice and injected 2 × 10^6^ cells through tail vain into BALB/c recipient mice. After inhalation of 100 µg OVA in the presence or absence of 20 µg lunasin for 3 days, mediastinal lymph nodes and lungs were harvested and made into single cell suspension. Cells were stained with CD4, KJ1.26 and Foxp3 and analyzed by flow cytometry. Intranasal lunasin treatment slightly increased antigen-specific (CD4^+^KJ1.26^+^) iTregs (Foxp3^+^) in the mediastinal lymph nodes without reach significance (Fig. 5A). However, antigen-specific iTregs in the lungs of lunasin treated mice were significantly higher and more than doubled than that of OVA only treated mice (Fig. 5B). The increased accumulation of antigen-specific iTregs in the lungs of lunasin treated mice were maintained 7 days (Fig. 5C) and 14 days (Fig. 5D) after the last intranasal treatment. The Foxp3^+^ DO11.10 T cells were mostly CD25^+^ and many were IL-10^+^ (S1 Fig.). The Foxp3^+^ DO11.10 T cells also expressed high levels of CTLA-4, which is necessary for immune suppression and prevention of in vivo autoimmunity [29, 30], suggesting these antigen-specific T cells are functional Tregs.

## Discussion

The global prevalence of asthma and allergy has increased over the past 2–3 decades especially within the developed in concert with growing clinical and socioeconomic burden [31]. Prolonged use of inhaled corticosteroids, especially at high doses, may cause systemic or local side effects [32]. In this report, we demonstrate that inhaled soybean peptide lunasin is efficient in alleviating allergic airway inflammation in both OVA+Alum and OVA+LPS sensitized mice with significantly reduced Th2 cytokine expression, inflammatory infiltration, and goblet cell metaplasia.

The NF-κB pathway is the master regulator of both innate and adaptive immune responses in a wide variety of cell types. Activation of NF-κB pathway leads to transcription of genes important in cell survival, proliferation and inflammation, and has been demonstrated to play a cardinal role in allergic airways disease [33]. Mice that lacked p50 or c-Rel subunits of NF-kB were protected from the development of allergic airway disease [34, 35]. Several studies suggest lunasin is capable of suppressing activation of NF-κB in both murine and human macrophages and expression of pro-inflammatory cytokines and mediators, likely through its interaction with the αVβ3 integrin [18, 20]. The factor that lunasin also interacts with α5β1 integrin to suppress NF-κB in colon cancer cells [15] suggest that, in addition to macrophages, lunasin can exert its anti-inflammatory effect on airway epithelial cells and bronchial fibroblasts as the α5β1 integrin—NF-κB pathway is operating in these cell types [36, 37].

In the asthmatic airways, various cells are activated to release cytokines, chemokines and chemicals including reactive oxygen and nitrogen species to cause inflammation or amplify inflammation. Recent evidence indicates that endogenous reactive oxygen and nitrogen species are responsible for the airway inflammation, and that the disequilibrium of the airway reducing state is a determinant of asthma severity [38]. Studies show that lunasin is a potent scavenger of peroxyl and superoxide radicals [39]. Lunasin significantly reduces intracellular reactive oxygen species (ROS) concentration in macrophages stimulated by LPS [19] and human Caco-2 cells challenged by hydrogen peroxide and tert-butylhydroperoxide [39]. Therefore, in addition to its anti-inflammatory activities through suppression NF-κB pathway, lunasin as a therapeutic agent also possesses antioxidant activities to further dampen allergic airway inflammation as seen in our studies.

While effective at controlling symptoms, current therapies for asthma and allergy do not change the chronic course of disease [3]. In contrast to the drug treatment that offers temporary symptomatic relief, allergy immunotherapy is unique in altering the clinical course of allergic diseases. One of the underlying mechanisms for allergy immunotherapy is the generation of allergen-specific iTregs [40, 41]. Our results that lunasin stimulates antigen-specific iTreg generation suggest that lunasin might be used as an adjuvant to enhance the efficacy of allergy immunotherapy, with added benefits of its anti-inflammatory activity to lower the risks associated with allergy immunotherapy.

It is also worth mentioning that systematic administration of lunasin (intraperitoneal injection) at the time of sensitization can prevent OVA+LPS induced airway sensitization so that airway inflammation is significantly reduced upon OVA challenge. Since lunasin can be detected in the plasma after soybean protein ingestion [42], an association study of consumption of soybean products and prevalence of sensitization against common allergens would be of clinical relevance.

## Supporting Information

S1 Fig Characterization of OVA-specific regulatory T cells in the mediastinal lymph nodes. Naive CD4^+^ T cells were purified from Foxp3^eGFP^ DO11.10 mice using MACS (Miltenyi). 2 × 10^6^ cells were adoptively transferred into wild type BALB/c mice and the recipients were treated intranasally with 100 µg OVA in the presence or absence of 20 µg lunasin. 14 days after the last treatment, mediastinal lymph nodes (MedLN) were collected for analysis. (A) Expression of CD25 and Foxp3 by the DO11.10 T cells. (B) Expression of IL-10 (intracellular staining) by Foxp3^+^ DO11.10 T cells. (C) Expression of CTLA-4 (intracellular staining) by Foxp3^+^ and Foxp3^-^ DO11.10 T cells.
(PDF)
