# Supplementary material for: Lunasin Alleviates Allergic Airway Inflammation while Increases Antigen-Specific Tregs
Source: PLoS One. 2015 Feb 3;10(2):e0115330. doi: 10.1371/journal.pone.0115330 (PMC4315393; doi:10.1371/journal.pone.0115330)
Supplement: S1 Fig — Naive CD4+ T cells were purified from Foxp3eGFP DO11.10 mice using MACS (Miltenyi). 2 × 10^6^ cells were adoptively transferred into wild type BALB/c mice and the recipients were treated intranasally with 100 µg OVA in the presence or absence of 20 µg lunasin. 14 days after the last treatment, mediastinal lymph nodes (MedLN) were collected for analysis. (A) Expression of CD25 and Foxp3 by the DO11.10 T cells. (B) Expression of IL-10 (intracellular staining) by Foxp3+ DO11.10 T cells. (C) Expression of CTLA-4 (intracellular staining) by Foxp3+ and Foxp3- DO11.10 T cells. (PDF) [file pone.0115330.s001.pdf]

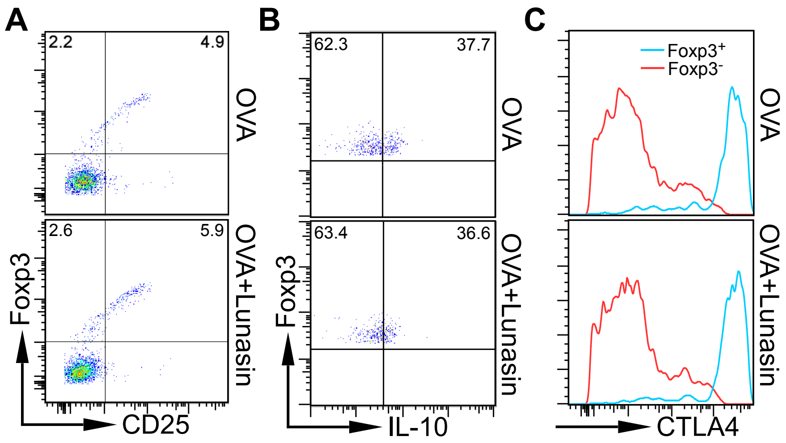

Figure S1. Characterization of OVA-specific regulatory T cells in the mediastinal lymph nodes.

Naive $CD4^+$ T cells were purified from $Foxp3^{eGFP}$ DO11.10 mice using MACS (Miltenyi). 2×10^6 cells were adoptively transferred into wild type BALB/c mice and the recipients were treated intranasally with 100 μg OVA in the presence or absence of 20 μg lunasin. 14 days after the last treatment, mediastinal lymph nodes (MedLN) were collected for analysis. (**A**) Expression of CD25 and Foxp3 by the DO11.10 T cells. (**B**) Expression of IL-10 (intracellular staining) by $Foxp3^+$ DO11.10 T cells. (**C**) Expression of CTLA-4 (intracellular staining) by $Foxp3^+$ and $Foxp3^-$ DO11.10 T cells.
